# Vitamin D May Increase the Number of CD4-CD8+ NKT-like Cells in Humans—A Novel Insight into Potential Immunomodulatory Action

**DOI:** 10.3390/nu17203216

**Published:** 2025-10-14

**Authors:** Emilia Adamska-Fita, Przemysław Wiktor Śliwka, Bartłomiej Stasiak, Małgorzata Karbownik-Lewińska, Magdalena Stasiak

**Affiliations:** 1 Department of Endocrinology and Metabolic Diseases, Polish Mother’s Memorial Hospital—Research Institute, 93-338 Lodz, Poland; emila0079@gmail.com (E.A.-F.); p.sliwka87@gmail.com (P.W.Ś.); malgorzata.karbownik-lewinska@umed.lodz.pl (M.K.-L.); 2 Department of Endocrinology and Metabolic Diseases, Medical University of Lodz, 93-338 Lodz, Poland; 3 Institute of Information Technology, Lodz University of Technology, 93-005 Lodz, Poland; bartlomiej.stasiak@p.lodz.pl

**Keywords:** NKT-like cells, CD4-CD8+ NKT-like cells, vitamin D, 25-hydroxycholecalciferol, immunomodulation, autoimmune disorders, immune response, cancer

## Abstract

**Background:** Vitamin D has a significant role in immune system regulation due to its profound impact on various immune cells, including Natural Killer T-like (NKT-like) cells. While previous studies have explored the effects of vitamin D on the overall NKT-like cell population, detailed investigations into its impact on specific NKT-like subpopulations are lacking. This study aimed to analyze the correlation between vitamin D levels and NKT-like cell subpopulations (CD4+CD8+; CD4-CD8+; CD4+CD8-; CD4-CD8-) in peripheral blood collected from patients without diseases that can influence vitamin D and/or calcium levels. **Methods:** Peripheral blood mononuclear cells (PBMCs) were isolated from 86 patients. NKT-like cells were separated from PBMCs using a CD3+ CD56+ NKT cell isolation kit and a magnetic bead separator. Flow cytometry (FC) was applied in order to evaluate the distribution of NKT-like cell subpopulations. **Results:** A significant positive correlation between vitamin D levels and the CD4-CD8+ NKT-like cell population, particularly the CD4-CD8high subtype was found. Importantly, this correlation was independent of calcium levels, emphasizing the unique impact of vitamin D on CD4-CD8+ NKT-like cells. **Conclusions:** Our findings suggest that vitamin D concentrations may influence the distribution of NKT-like cell subpopulations in peripheral blood, although further evidence is necessary to confirm this observation. These novel results provide a foundation for elucidating the mechanism underlying the effect of vitamin D on the immune system and may contribute to future therapeutic strategies targeting CD4-CD8+ NKT-like cells in immune and oncological disorders.

## 1. Introduction

The role of vitamin D in human physiology has been a focus of scientific interest for over 100 years. The earliest studies linking vitamin D to the prevention of rickets date back to the early 20th century. However, the natural form of vitamin D was not discovered until 1936, and its active form, 1,25-dihydroxyvitamin D (1,25(OH)2D), was identified in 1971 [1].

Vitamin D plays a dual role, functioning both as a vitamin and a hormone, depending on its form and function. It is considered a vitamin because vitamin D is obtained from external sources and is essential for calcium and phosphate homeostasis and bone mineralization. After undergoing 25- and 1α-hydroxylation in the liver and kidneys to its active form (calcitriol), it functions as a hormone, binding to the vitamin D receptor (VDR) in the cellular nucleus [2]. Upon the binding of calcitriol to VDR, a heterodimer is formed with the retinoid X receptor (RXR); this binds to DNA sequences known as vitamin D response elements (VDREs) in the promoter regions of target genes, leading to increased or decreased gene transcription [3]. The VDR belongs to the steroid hormone receptor superfamily and is located in various tissues, including the kidneys, skeletal system, digestive system, endocrine system, and nervous system, as well as being expressed in immune cells such as monocytes, macrophages, T lymphocytes, B lymphocytes, Natural Killer (NK) cells, and Natural Killer T (NKT) cells [4,5,6,7,8,9,10,11,12].

Previously described in the literature as Natural Killer T (NKT) cells, NKT-like cells form a distinct and diverse group of T lymphocytes that combine functional and phenotypic features of T cells (i.e., T cell receptor (TCR)) and NK cells (i.e., cluster of differentiation (CD)56). The primary classification of NKT cells, which was based on CD3 and CD56 antigen expression, has been changed according due to the finding that CD56 is non-specific and can be detected on a broader spectrum of T lymphocytes, particularly among activated γδ T cells and within populations of αβ T cells [13,14,15]. Cells expressing CD56 are generally known to have a more activated phenotype that is frequently connected with cytotoxic potential and the partial sharing of functional properties [16]. NKT-like cells act as a bridge between innate and adaptive immunity, as they possess cytotoxic properties due to the production of perforin and granzyme, and produce a broad range of cytokines regulating inflammatory responses through direct and indirect interactions with other cells of the immune system [15,17]. Unlike other subpopulations of NKT-like cells, invariant NKT (iNKT) cells are very rare in peripheral blood; these cells are characterized by recognizing lipid antigens presented by the non-classical MHC molecule CD1d [18,19]. Advances in immunology, with particular emphasis on more detailed cell identification approaches, have resulted in continual reassessment of iNKT and NKT-like cell categorization [13,14]. Based on the expression of CD4 and CD8, iNKT-cells and NKT-like cells can be divided into four subpopulations: CD8+CD4-, CD4+CD8-, CD4-CD8- (double negative; DN), and CD4+CD8+ (double positive; DP) [20]. The CD4-CD8+ subpopulation predominantly releases Th1 cytokines (interferon gamma—IFN-γ; tumor necrosis factor alfa—TNF-α) and exhibits cytotoxic activity, thereby participating in antiviral, antibacterial, and antitumor responses [21].

Numerous studies have evaluated the impact of vitamin D on cancer and autoimmune and infectious diseases in both human and animal models [1,22]. The potential of adequate vitamin D levels to produce a protective effect in autoimmune diseases has been postulated [23]. Vitamin D–VDR signaling is also essential for proper NKT-like cells functioning. In murine models, vitamin D–VDR signaling has been identified as a critical point of iNKT cell maturation and thymic development. Evidence from prenatal vitamin D deficiency showed that improper fetal exposure resulted in epigenetic modifications that persistently reduce iNKT cell populations [11,12]. Undeniably, vitamin D modulates immune system function, including interaction with NKT-like cells [7,9,10,11,12]. Previous studies, including our own research on the impact of type 2 diabetes mellitus (T2DM) on NKT-like cell populations, have emphasized the vulnerability of NKT-like cells to metabolic disorders [24,25,26].

Nevertheless, specific effects of vitamin D on NKT-like subpopulations remain insufficiently characterized. The aim of our study was to analyze the correlation between serum vitamin D levels and the distribution of NKT-like cell subpopulations in the peripheral blood of patients without parathyroid gland disorders or other diseases that could affect vitamin D or calcium homeostasis.

## 2. Materials and Methods

### 2.1. Patients

The research included 86 patients of Caucasian origin (68 women and 18 men) diagnosed in the Department of Endocrinology with thyroid nodular disease, in whom the benign character of the thyroid nodules was cytologically confirmed. Inclusion criteria were as follows: adult patients; benign thyroid nodular disease confirmed cytologically with no other thyroid disease; patient consent for participation in the study; absence of any of the exclusion criteria. The group was selected from outpatient clinic patients living in the same geographical area. Exclusion criteria included presence of any other acute or chronic disease that may influence either vitamin D and/or calcium levels or immune system response and function. Similarly, patients who used any medication that may influence the result were excluded from the study. Seventy-one of the patients (82.56%) took cholecalciferol in doses ranging from 1000 to 4000 IU/day. Other medications used by the patients included magnesium supplements (8%) and omega 3 acid supplements (5%). The mean age of participants was 58.09 ± 14.07 years. The clinical characteristics of the study group, including vitamin D and calcium levels, are presented in Table 1.

### 2.2. Biochemical Analysis

For the evaluation of serum vitamin D levels, peripheral venous blood samples were obtained from patients at 6:00 a.m. under fasting conditions, regardless of the time of year. Serum concentrations of vitamin D and total calcium were assessed using the electrochemiluminescence immunoassay (ECLIA) method on the Cobas e601 analyzer (Roche Diagnostics, Indianapolis, IN, USA).

### 2.3. NKT-like Cell Isolation

Peripheral blood samples (2 × 4.9 mL) were collected into EDTA tubes (Sarstedt, Nümbrecht, Germany) via venipuncture. Peripheral blood mononuclear cells (PBMCs) were isolated through gradient centrifugation at 400× *g* for 30 min using Histopaque^®^-1077 (Thermo Fisher Scientific, Waltham, MA, USA).

NKT-like cells were separated from PBMCs using a CD3+ CD56+ NKT cell isolation kit (No. 130-093-064) and a magnetic bead separator (Miltenyi Biotec, Bergisch Gladbach, Germany). Sequential isolation was performed in two steps following the manufacturer’s instructions: first, PBMCs were initially depleted of NK cells and monocytes, which were indirectly magnetically labelled using a cocktail of biotin-conjugated antibodies and Anti-Biotin MicroBeads. CD3+ CD56+ NKT-like cells are further enriched in the second step of magnetic separation.

### 2.4. Flow Cytometry (FC)

To characterize the distribution of NKT-like cell subpopulations, flow cytometry (FC) was applied. Cells were labelled using fluorochrome-conjugated monoclonal antibodies directed against human CD3 (APC, clone UCHT1) and CD56 (PE-Cy7, clone B159), both obtained from Becton Dickinson (Franklin Lakes, NJ, USA), to enable identification of NKT-like cells. In addition, an anti-Vα24JαQ TCR chain antibody (PE, clone 6B11) was used to quantify the invariant NKT (iNKT) fraction, which was found to be very low (~1.2%). Since neither αβ nor γδ T-cell receptors were assessed on the isolated cells, all subsequent analyses were carried out on the heterogeneous CD3+CD56+ NKT-like population, in line with current conventions. For subset discrimination, further staining was performed with antibodies recognizing CD4 (FITC, clone SK3) and CD8 (PerCP, clone SK1), also supplied by Becton Dickinson. To confirm the specificity of staining, isotype-matched controls were included in every experiment. Flow cytometric acquisition was performed on a BD FACSCanto II cytometer (Becton Dickinson, NJ, USA), and data processing was carried out with the manufacturer’s analysis software (BD FACSDiva Software 6.1.2).

**Figure 1:**
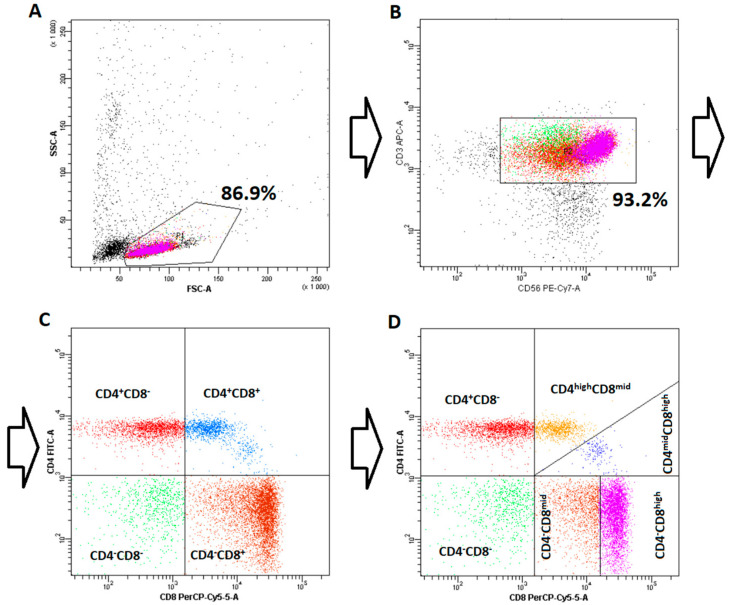
An example of flow cytometry (FC) plots demonstrating the gating strategy used for cell analysis. (**A**) PBMC-derived population post-magnetic enrichment; (**B**) CD3^+^CD56^+^ NKT-like cells following FC purification; (**C**) Primary gating scheme for initial NKT-like cell identification; (**D**) Refined gating strategy for distinguishing NKT-like cell subpopulations.

Initially, NKT-like cells were divided into four main subpopulations, CD4-CD8- (double negative), CD4-CD8+, CD4+CD8-, and CD4+CD8+ (double positive), according to the classification described by Montoya et al. [20], which was originally developed for iNKT cells. Further analysis led to the discovery of clear heterogeneity in both CD8+ subpopulations, leading to advanced subdivision of CD4+CD8+ and CD4-CD8+ subpopulations into CD4highCD8mid/CD4midCD8high and CD4-CD8mid/CD4-CD8high, respectively. The detailed gating strategy is presented in Figure 1.

### 2.5. Statistical Analysis

Analysis of the correlation between vitamin D levels and the percentage of individual subpopulations of NKT-like cells—including both the main four subpopulations (henceforth denoted as “types”) and the results of the subsequent subdivisions (mid/high subtypes)—was performed on the basis of Pearson’s linear correlation coefficient and Spearman’s rank correlation coefficient. For the analysis of statistically significant differences between patient groups, Student’s *t*-test and the Mann–Whitney U-test were applied, while the normality of parameter distribution within groups was controlled using the Shapiro–Wilk test. For the statistical analysis and plot generation, scipy.stats and matplotlib libraries (version 1.10.1 and 3.7.5, respectively) were used. Patients were clustered into groups with the Expectation-Maximization (EM) algorithm implemented in the Waikato Environment for Knowledge Analysis (Weka, version 3.6.14).

### 2.6. Ethics Procedures

After receiving a detailed explanation of the purpose and course of the study, all patients signed written informed consent. The study was approved by the Ethics Committee of the Polish Mother’s Memorial Hospital—Research Institute in Łódź, Poland (approval code: 41/2021).

## 3. Results

The mean level of vitamin D was 31.7 ±16.87 ng/mL, median 28.7 ± 10.57 ng/mL. The mean level of calcium was 2.33 ± 0.15 mmol/L), median 2.33 ± 0.11 mmol/L. No correlation between vitamin D and calcium level for the whole group was found: Pearson’s correlation coefficient was equal to 0.179 (*p*-value = 0.102), while Spearman’s ρ was 0.183 (*p*-value = 0.093).

In the first part of the study, we analyzed correlations of the main NKT-like cell types (i.e., CD4-CD8+, CD4-CD8-, CD4+CD8+, CD4+CD8-) with vitamin D level. A significant positive correlation between vitamin D level and CD4-CD8+ NKT-like cells was demonstrated. Pearson’s correlation coefficient was equal to 0.312 (*p*-value = 0.003), while Spearman’s ρ was 0.225 (*p*-value = 0.006) (Table 2). In the case of other NKT-like cell types, no significant correlation with vitamin D was found (Table 2). Additionally, a slight positive correlation between CD4-CD8+ NKT-like cells and calcium level was demonstrated, with statistical significance reached only for Spearman’s rank correlation (ρ = 0.293, *p*-value 0.039) (Table 2).

In the next part of the study, we analyzed correlations of the subtypes of each type of NKT-like cell (i.e., CD4highCD8mid, CD4midCD8high, CD4-CD8mid, CD4-CD8high) with vitamin D and calcium, respectively. The analysis revealed a significant positive correlation between vitamin D and the CD4-CD8high subtype (Pearson’s ρ = 0.328, with *p*-value 0.004; and Spearman’s ρ = 0.262, with *p*-value 0.021).

**Figure 2:**
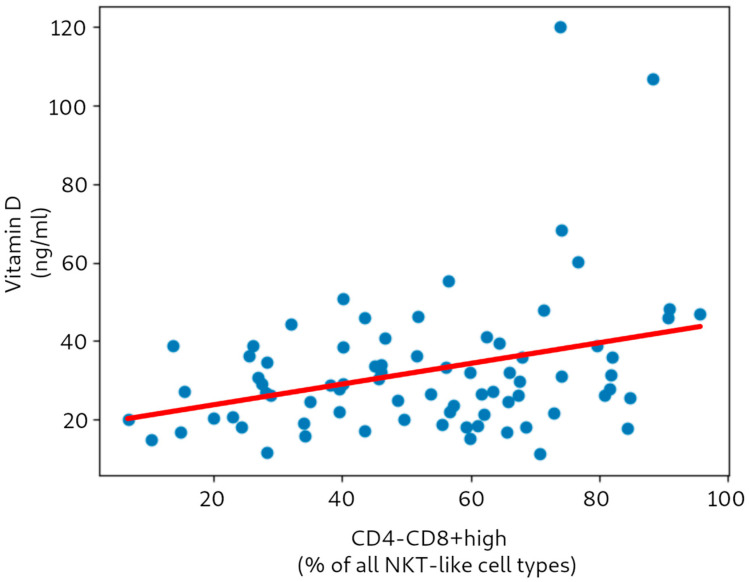
Correlation and linear regression between vitamin D level and the CD4-CD8high subtype (*n* = 77).

Figure 2 presents the correlation between vitamin D level and the CD4-CD8high NKT-like cell subtype. It should be stressed that removal of the two prominent outliers (with increased vitamin D levels due to excessive supplementation) did not greatly influence the obtained correlation coefficients—for either the types or subtypes of NKT-like cells. For example, the value of Pearson’s ρ for vitamin D vs. CD4-CD8+ after outlier removal was 0.304 (*p* 0.005).

In the case of other NKT-like cell subtypes, including the CD4-CD8mid subtype, no significant correlation with vitamin D was found (Table 3). No correlation between the CD4-CD8high subtype and calcium level was found, although significant correlation was demonstrated between the CD4-CD8mid subtype and calcium level (Table 3).

The correlations in Table 3 were calculated for the raw percentages of the NKT-like cells of each subtype. For example, if the CD4+CD8+ type constituted 40% of all NKT-like cells of a patient, divided equally into CD4highCD8mid and CD4midCD8high, both these subtypes were represented as 20% and 20%, respectively. We additionally tested a hypothesis that it is not the absolute percentage but the relative proportion of the subtypes that should be taken into consideration (so both subtypes would be represented as 50% and 50% in our example, irrespective of the absolute percentage of CD4+CD8+ cells). This hypothesis was not confirmed, as demonstrated in Table 4.

**Figure 3:**
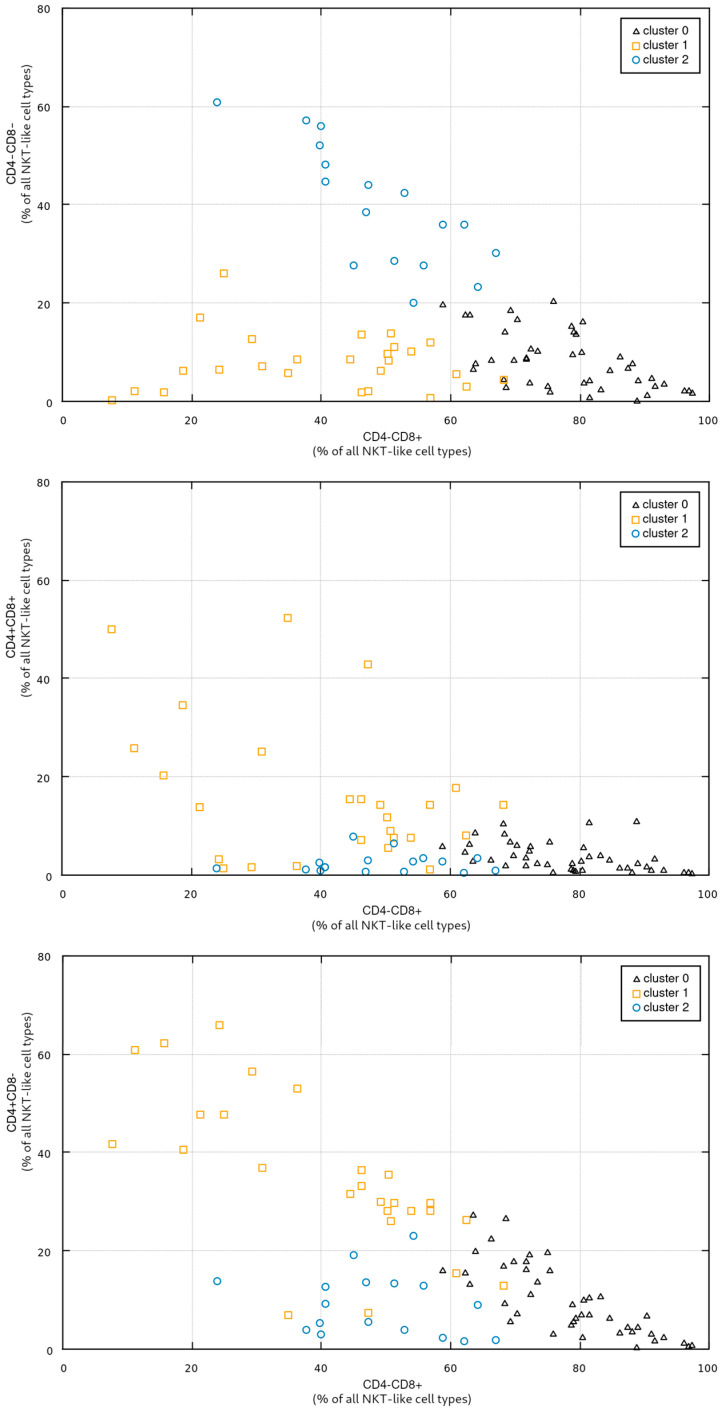
Visualization of NKT-like type clusters (from top to bottom: CD4-CD8- vs. CD4-CD8+; CD4+CD8+ vs. CD4-CD8+; CD4+CD8- vs. CD4-CD8+); number of cases in clusters 0, 1, and 2 was 43, 26, and 17, respectively.

In the third step of the study, in order to investigate potential relationship between the proportions of individual NKT-like cell types and vitamin D levels, we clustered the patients into three groups using a standard EM approach (Expectation-Maximization [27,28]). The clustering was performed in a four-dimensional space spanned by all four CD4/CD8 NKT-like cell types (CD4+CD8+, CD4+CD8-, CD4-CD8+, CD4-CD8-). Figure 3 shows the obtained groups in some selected subspaces (i.e., in all subspaces involving the CD4-CD8+ NKT-like cell type). The cardinality of the groups, the coordinates of their respective centroids, and standard deviations along individual axes are presented in Table 5.

Figure 3 presents the cluster distribution, with cluster 0 containing the patients with high a percentage of CD4-CD8+ NKT cells; cluster 1 grouping the patients with a low-to-medium percentage of CD4-CD8+, low CD4-CD8-, and rather high CD4+CD8-; and cluster 2 grouping patients with low CD4+CD8+ and CD4+CD8-, medium CD4-CD8+, and high CD4-CD8-.

An important conclusion is that the clustering procedure (assuming the fixed number of three groups) proved quite stable, yielding very similar results for different initial conditions and for a different clustering algorithm based on the standard k-means approach [29].

The results of clustering are presented in Table 6. As for the choice of the test type, we additionally tested for the normality of the vitamin D distribution in each cluster. It revealed that it deviates from normal pdf in cluster 0; therefore, the non-parametric Mann–Whitney test should be used in the first two cases (cluster 0 vs. 1 and 0 vs. 2), which means that the only comparison result that should be considered statistically significant is cluster 0 (involving the highest percentage of CD4-CD8+ NKT-like cells) vs. cluster 1 (involving the lowest percentage of CD4-CD8+ NKT-like cells and the highest percentage of CD4+ NKT-like cells) (*p*-value = 0.027) (Table 6).

To further demonstrate the relationship between vitamin D and CD4-CD8+ NKT-like cells, we divided the patients into two groups by thresholding the CD4-CD8+ percentage value. Using the threshold of 50%, we obtained the two groups presented in Table 7.

A statistically significant difference between the groups was found (Student’s *t*-test statistic t = 3.185; *p*-value = 0.002). As the vitamin D distribution in the first group deviated significantly from normal pdf, we repeated the analysis with a Mann–Whitney test, which also confirmed statistical significance (U = 1018; *p*-value = 0.015). The effect size (rank-biserial correlation) computed for the U statistic is 0.335.

## 4. Discussion

Research conducted so far emphasizes that vitamin D plays a pivotal role in the development, function, and regulation of NKT-like cells, a crucial component of the immune system [11,12]. Current studies have focused on correlations between vitamin D levels and the overall NKT-like cell population or iNKT cells specifically [11,12]. However, detailed results on the interactions between NKT-like cell subpopulations and vitamin D are still lacking.

To the best of our knowledge, our study is the first one to analyze the correlation between vitamin D level and NKT-like cell subpopulations in the peripheral blood of patients without disorders that may influence calcium and/or vitamin D levels. The lack of correlation between vitamin D and total calcium in the study cohort further confirmed that the results were not influenced by any calcium/vitamin D-related disturbances. Previous studies primarily classified NKT cells into two or three subpopulations: CD4+, CD4-, and CD4-CD8- (double negative) [30,31,32,33]. Using fluorescence-activated cell sorting, we categorized NKT-like cells into four subpopulations, CD4+CD8-, CD4-CD8+, CD4-CD8- (double negative, DN), and CD4+CD8+ (double positive, DP), following the classification applied by Montoya et al. [20]. A more detailed analysis revealed significant heterogeneity within the CD8+ subpopulations. Therefore, we further performed an advanced stratification of the CD4+CD8+ and CD4-CD8+ subpopulations. Specifically, CD4+CD8+ and CD4-CD8+ cells were subdivided into CD4highCD8mid/CD4midCD8high and CD4-CD8mid/CD4-CD8high, respectively. Such a stratification of NKT-like cell subpopulations has not been presented in the literature before, but seems highly important for detailed knowledge on the actual role of NKT-like cells, due to the potentially different functions of the NKT-like cell subpopulations, which may not be demonstrated for the whole NKT-like population or even for the CD4+CD8-, CD4-CD8+, CD4-CD8-, CD4+CD8+ subpopulations. Our study involved detailed subpopulations and subpopulations of the subpopulations to confirm the observed correlation of CD4-CD8+ with vitamin D level.

The current analysis identified a positive correlation exclusively between vitamin D levels and the number of CD4-CD8+ cells. No other significant correlations were observed between vitamin D and the other NKT-like cell subpopulations. To further confirm this correlation, we performed clustering in a four-dimensional space spanned by all four CD4/CD8 NKT cell types (CD4+CD8+, CD4+CD8-, CD4-CD8+, CD4-CD8-). This approach allowed us to demonstrate a significant difference between vitamin D levels in patients belonging to the cluster with the highest percentage of CD4-CD8+ NKT-like cells and the ones belonging to the cluster with the highest percentage of CD8-negative NKT-like cells. As our observation is novel and constitutes an important step in the development of knowledge on vitamin D–immune system interplay, we decided to apply a third different approach to confirm the results. In this analysis, the patients were divided into two groups by thresholding the CD4-CD8+ percentage value at 50%. This approach further confirmed our previous results, as a significantly higher vitamin D level was detected in a group of patients with a domination (above 50%) of CD4-CD8+ cells. Modest simultaneous correlation of total calcium with CD4-CD8+ demonstrated in the first analysis was not confirmed in the further steps of the study, which leads to presumption that the correlation between vitamin D and NKT-like cell subpopulations is calcium-independent, at least in patients without diseases influencing calcium levels.

Our results are difficult to compare with other studies as, to date, no other results regarding the correlation between vitamin D and NKT-like cell subpopulations have been published. Only studies analyzing the total NKT cell population or iNKT cell population are available [11,12,34].

Taking into account the novelty of our results, we decided to perform even more detailed analysis. Initially, NKT-like cells were subdivided into four main subpopulations: CD4-CD8-, CD4-CD8+, CD4+CD8- and CD4+CD8+. However, further analysis led to the discovery of clear heterogeneity in both of the CD8+ subpopulations, leading to advanced subdivision of CD4+CD8+ and CD4-CD8+ subpopulations into CD4highCD8mid/CD4midCD8high and CD4-CD8mid/CD4-CD8high cells, respectively. This subclassification of NKT-like cell subpopulations has never been described before. Our investigation revealed a significant positive relationship between vitamin D and the CD4-CD8high subtype, but no significant associations were found between vitamin D and the other NKT-like cell subtypes, including the CD4-CD8mid subtype, which further indicates the possible exclusive influence of vitamin D on NKT-like cell subpopulations with high CD8 expression. As, conversely, calcium levels were not correlated with CD4-CD8high subtype but with CD4-CD8mid subtype, we can consider this observation as further proof that vitamin D’s impact on NKT-like cells is calcium-independent. Studies exploring the connection between NKT-like cells and calcium levels are scarce and primarily concentrated on intracellular calcium regulation mechanisms and their influence on NKT cell activity [35]. Direct studies assessing the correlation between serum calcium concentration and NKT-like cell numbers are lacking.

As mentioned above, our results cannot be directly compared to other authors’ observations due to lack of similar analyses in the literature. However, the available results of studies in the field of vitamin D and the immune system provide data that may explain the importance of our findings. Bychinin et al. reported a positive association between vitamin D levels and NKT cells in COVID-19 patients admitted to the intensive care unit (ICU). Their study demonstrated that vitamin D supplementation significantly increased the total number of NKT cells in peripheral blood compared to the placebo group [34]. Additionally, based on murine models, it was revealed that the vitamin D–VDR signaling pathway is essential for the proper maturation and maintenance of adequate NKT cell populations [11,12]. These studies did not analyze the CD4CD8-subpopulations of NKT-like cells, but their results are crucial in underscoring the critical role of sufficient vitamin D levels in the development and maintenance of NKT-like cells. At present, there is a lack of research directly investigating the molecular mechanisms by which vitamin D regulates NKT-like cells. The available literature focuses predominantly on vitamin D–VDR signaling in iNKT cell development [11,12]. Yue et al. demonstrated that the transcriptional co-activator Med1, which interacts with VDR, is essential for iNKT cell development [36]. The analysis of molecular mechanisms was not the aim of our study, and further investigations are required to explain this association.

The impact of vitamin D on the immune system is currently being thoroughly analyzed. The interaction between vitamin D and NKT-like CD4-CD8+ cells is crucial for maintaining human health. Vitamin D has been shown to have a protective effect on many pathological conditions, including allergic diseases such as allergic rhinitis and atopic dermatitis [37,38,39,40]. Animal model studies suggest a correlation between serum vitamin D levels and inflammatory markers [37,41]. NKT-like cells are critical regulators of immune responses in allergic diseases due to their ability to secrete both pro-inflammatory and anti-inflammatory cytokines that modulate allergic reactions [15]. IFN-γ, primarily produced by the NKT-like CD4-CD8+ subpopulation, antagonizes Th2 cytokines like IL-4 and IL-13, promoting a more balanced immune response and reducing tissue damage [42]. Our results may provide a potential key mechanism regarding vitamin D’s action on NKT-like cells in allergic reactions, i.e., attenuating the Th2-related response by increasing the number CD4-CD8+ NKT-like cells. This hypothesis requires further study to confirm such a causality.

Furthermore, vitamin D has been shown to exert protective effects against autoimmune diseases. The immunomodulatory effect of vitamin D involves, among others, modification of the profile of cytokines produced by NKT-like cells. Increasing the production of anti-inflammatory cytokines can suppress excessive immune responses and tissue injury. The VITAL study revealed that long-term vitamin D supplementation reduced the risk of all autoimmune diseases by 22% compared to the placebo group [23]. Moreover, in an experimental autoimmune encephalomyelitis (EAE) mouse model, Waddell et al. demonstrated that interactions between vitamin D and NKT cells are essential for protection against EAE. In NKT-deficient mice, the protective effects of vitamin D were attenuated [43]. A reduction in the amount NKT-like cells has also been consistently noted in other autoimmune diseases. Zhou et al. [44] found that patients with primary Sjögren’s syndrome have significantly diminished circulating NKT-like cells, and this phenomenon correlates with higher disease severity. Also, Lin et al. [45] revealed that NKT-like cell numbers are reduced in systemic lupus erythematosus patients and that NKT-like cells are functionally impaired, with decreased cytotoxicity and IFN-γ production.

Through its influence on gene transcription, vitamin D plays a crucial role in regulating the growth, differentiation, and apoptosis of human cells [46]. Some epigenetic studies have shown that VDR is overexpressed in colorectal, prostate, and ovarian cancers, and increased VDR expression on tumor cells may be associated with a more favorable response to treatment [47,48,49,50,51]. A meta-analysis conducted by Keum et al. suggested that vitamin D reduces cancer mortality [52]. While the underlying mechanism remains unclear, it is likely influenced by the effects of vitamin D on the various types of immune cells, including T lymphocytes, NK cells, macrophages, dendritic cells (DCs), and CD4-CD8+ NKT-like cells. NKT-like CD4-CD8+ cells interact with tumor cells by releasing cytolytic proteins and producing of a wide range of cytokines, such as IFN-γ and TNF-α, affecting other immunocompetent cells, including DCs, T lymphocytes, and NK cells [15,17,53]. Furthermore, IFN-γ, when secreted in significant amounts by CD4-CD8+ NKT-like cells, inhibits angiogenesis, thereby exhibiting strong antitumor activity [51,54]. Alves et al. demonstrated that NKT-like cells can be expanded ex vivo from patients with ovarian cancer, where they have strong cytotoxicity against tumor cells [55]. Conversely, Yuen et al. [56] showed that tumor-infiltrating NKT-like cells in hepatocellular carcinoma upregulate inhibitory receptors, indicating suppression of effector function within the tumor microenvironment. These findings support our results, suggesting that vitamin D may enhance the antitumor role of CD4-CD8+ NKT-like cells, particularly regarding the novel CD8high subtype.

Vitamin D and NKT-like cells are synergistic elements of antimicrobial defense, with their combined actions strengthening the immune response and modulating its course in a manner beneficial to the organism. Vitamin D plays a significant role in antibacterial and antiviral responses by stimulating the production of antimicrobial peptides, such as cathelicidins and alpha- and beta-defensins, and enhancing the cytotoxic activity of NK cells and macrophages [22,51]. Additionally, vitamin D stimulates CD4-CD8+ NKT-like cells to produce IFN-γ and other cytokines, which in turn increase the activation of macrophages and T lymphocytes—key players in the antimicrobial immune response [15,17]. Moreover, in study conducted by Kokordelis et al. [57], NKT-like cells have been shown to contribute to antiviral defense in patients with acute hepatitis C. While the antimicrobial properties of vitamin D have been extensively studied, the findings remain inconsistent [1,22,58]. Research conducted by Liu et al. demonstrated that vitamin D promotes the expression of cathelicidin LL-37/hCAP-18 in human macrophages, offering protection against M. tuberculosis infection through the activation of TCR 2/1 and VDR [58]. On the other hand, the incidence of respiratory infections occurred at similar levels in placebo and vitamin D groups [1]. Further studies are required to assess the effect of vitamin D on the risk of occurrence and course of infectious diseases, but an adequate supply of vitamin D seems beneficial for the antimicrobial actions of NKT cells and other immune cells [58].

Vitamin D has a significant role in immune system regulation by influencing NKT-like cells, which affect allergic reactions, autoimmune disorders, cancers, and antimicrobial defense. Our study demonstrated a positive correlation between vitamin D and CD4-CD8+ NKT-like cells (further defined as CD4-CD8high) and may constitute the initial step for further explanation of immunomodulatory effect of vitamin D in several disorders and contribute to the future development of novel therapeutic approaches to treat a wide range of immunological and oncological disorders. However, the study has several limitations that should be taken into account. Firstly, the cohort was relatively small, restricted to Caucasian patients, and predominantly included females with benign thyroid nodules. Secondly, we did not analyze the functional properties of NKT-like cells with regard to the observed correlation with vitamin D. Moreover, the majority of participants (82.6%) were receiving vitamin D supplementation, and the impact of supplementation dose and duration on NKT-like cells subpopulations was not analyzed. However, we believe that analysis performed on a population with overt vitamin D deficiency may be less reliable, as different immune response disturbances may result from the vitamin D deficiency. We aimed to analyze whether vitamin levels in patients without overt deficiency may influence the profile of NKT-like cell types and subtypes. Therefore, the choice of a cohort in whom the supplementation was common was intentional.

## 5. Conclusions

Our study is the first to demonstrate a positive correlation between vitamin D and the number of CD4-CD8+ NKT-like cells, with a more detailed analysis emphasizing the particular effect regarding the CD4-CD8high subtype, which provided further evidence of the immunomodulatory properties of vitamin D. These findings highlight the critical role of the CD4-CD8+ NKT-like cell subpopulation in the interplay between vitamin D and the immune system. Further studies in larger, multicenter cohorts are warranted to confirm our results and explore the underlying molecular mechanisms. Emphasis should be put on assessment of the functional properties of NKT-like cells, including cytokine production and cytotoxic activity, to support the development of new, targeted therapies in immune and oncological disorders.

## Figures and Tables

**Table 1:** Clinical characteristics of the study group.

Parameter [unit]	Mean	SD	Median	Reference Range
Vitamin D [ng/mL]	31.7	16.87	28.7	30–50
Calcium	2.33	0.15	2.33	2.2–2.55
CRP [mg/L]	0.64	0.56	0.5	<1.0
ESR [mm/h]	10.94	19.29	2	<15
TSH [mIU/L]	1.41	1.61	1.03	0.27–4.2
FT4 [ng/dL]	1.28	0.31	1.25	0.93–1.7
Creatinine [mg/dL]	0.78	0.17	0.76	0.55–1.2
Glucose [mg/dL]	94.04	11.30	92	70–99
BMI [kg/m^2^]	25.29	3.86	24.58	<30

**Table 2:** Correlations of NKT-like cell type percentage with vitamin D and calcium levels.

NKT-like Cell Subpopulation	Mean ± Std		Correlation Parameter	Pearson’s ρ	*p* Value	Spearman’s ρ	*p* Value
CD4+CD8-	17.46% ± 15.68%	vs.	Vitamin D	−0.177	0.103	−0.185	0.089
CD4+CD8-	17.46% ± 15.68%	vs.	Calcium	−0.183	0.093	−0.247	0.022
CD4+CD8+	7.29% ± 10.05%	vs.	Vitamin D	−0.174	0.109	−0.203	0.061
CD4+CD8+	7.29% ± 10.05%	vs.	Calcium	−0.06	0.586	−0.152	0.165
CD4-CD8-	14.32% ± 14.58%	vs.	Vitamin D	−0.141	0.196	−0.182	0.093
CD4-CD8-	14.3%2 ± 14.58%	vs.	Calcium	−0.083	0.452	−0.037	0.736
CD4-CD8+	60.93% ± 21.57%	vs.	Vitamin D	0.312	0.003	0.225	0.006
CD4-CD8+	60.93% ± 21.57%	vs.	Calcium	0.212	0.051	0.293	0.039

**Table 3:** Correlations of NKT-like cell subtype percentage with vitamin D and calcium levels.

NKT Cell Subtype (Absolute Percentage)	Mean ± Std		Correlation Parameter	Pearson’s ρ	*p* Value	Spearman’s ρ	*p* Value
CD4highCD8mid	6.88% ± 10.26%	vs.	Vitamin D	−0.145	0.208	−0.202	0.079
CD4highCD8mid	6.88% ± 10.26%	vs.	Calcium	−0.113	0.33	−0.135	0.246
CD4midCD8high	1.04% ± 1.18%	vs.	Vitamin D	−0.123	0.289	0.013	0.909
CD4midCD8high	1.04% ± 1.18%	vs.	Calcium	0.103	0.378	0.04	0.73
CD4-CD8mid	8.04% ± 8.71%	vs.	Vitamin D	−0.055	0.635	0.022	0.85
CD4-CD8mid	8.04% ± 8.71%	vs.	Calcium	0.222	0.054	0.25	0.029
CD4-CD8high	52.64% ± 21.74%	vs.	Vitamin D	0.328	0.004	0.262	0.021
CD4-CD8high	52.64% ± 21.74%	vs.	Calcium	0.087	0.454	0.042	0.716

**Table 4:** Correlations of NKT-like cell subtype related proportion with vitamin D and calcium levels.

NKT-like Cell Subtype (Relative Proportion)	Mean ± Std		Correlation Parameter	Pearson’s ρ	*p* Value	Spearman’s ρ	*p* Value
CD4highCD8mid	69.36% ± 27.27%	vs.	Vitamin D	−0.022	0.848	−0.145	0.209
CD4highCD8mid	69.36% ± 27.27%	vs.	Calcium	−0.068	0.562	−0.124	0.284
CD4midCD8high	30.64% ± 27.27%	vs.	Vitamin D	0.022	0.848	0.145	0.209
CD4midCD8high	30.64% ± 27.27%	vs.	Calcium	0.068	0.562	0.124	0.284
CD4-CD8mid	14.06% ± 12.54%	vs.	Vitamin D	−0.129	0.265	−0.099	0.392
CD4-CD8mid	14.06% ± 12.54%	vs.	Calcium	0.173	0.135	0.175	0.13
CD4-CD8CD8high	85.94% ± 12.54%	vs.	Vitamin D	0.129	0.265	0.099	0.392
CD4-CD8CD8high	85.94% ± 12.54%	vs.	Calcium	−0.173	0.135	−0.175	0.13

**Table 5:** The parameters of the obtained clusters.

Cluster ID		0	1	2
Number of Patients		43	26	17
CD4+CD8-	mean	9.91	35.17	9.11
	std	7.32	15.62	6.01
CD4+CD8+	mean	3.72	16.10	2.68
	std	2.99	14.19	2.07
CD4-CD8-	mean	8.06	8.11	39.19
	std	5.74	5.80	12.37
CD4-CD8+	mean	78.32	40.63	49.03
	std	10.21	16.75	11.03

**Table 6:** The results of vitamin D level comparison between the clusters.

	Cluster 0 vs. Cluster 1	Cluster 0 vs. Cluster 2	Cluster 1 vs. Cluster 2
Student’s t	t = 2.729 (*p*-value = 0.008)	t = 2.049 (*p*-value = 0.045)	t = −0.624 (*p*-value = 0.537)
Mann–Whitney	U = 738 (*p*-value = 0.027)	U = 461.5 (*p*-value = 0.117)	U = 198.5 (*p*-value = 0.585)

**Table 7:** The characteristics of vitamin D parameters in patients with high (>50) and low (≤50) percentages of CD4-CD8+ NKT-like cells.

Group	Vitamin D								
CD4-CD8+	*N*	Mean	Std	IQR	Min	1st Quart.	Median	3rd Quart.	Max
>50%	61	34.38	18.77	14.90	11.20	24.60	30.00	39.50	120.00
≤50%	25	25.16	8.07	12.70	11.60	18.00	24.70	30.70	38.90

## Data Availability

The data presented in this study are not publicly available due to privacy restrictions but they are available on request from the corresponding author.

## Abbreviations

The following abbreviations are used in this manuscript:

1,25(OH)2D	1,25-dihydroxyvitamin D
α-GalCer	α-galactosylceramide
AITD	autoimmune thyroid disease
anti-Tg	thyroglobulin antibodies
anti-TPO	thyroid peroxidase antibodies
APCs	antigen presenting cells
CD	cluster of differentiation
COVID-19	coronavirus disease 2019
DCs	dendritic cells
DN	double negative
DP	double positive
EAE	experimental autoimmune encephalomyelitis
ECLIA	electrochemiluminescence immunoassay
EDTA	ethylenediamine tetraacetic acid
EM	Expectation-Maximization
FC	flow cytometry
ICU	intensive care unit
Il	interleukin
IFN-y	interferon gamma
iNKT	invariant Natural Killer T cells
MCH	major histocompatibility complex
mRNA	messenger ribonucleic acid
NK cells	Natural Killer cells
NKT cells	Natural Killer T cells
NKT-like cells	Natural Killer T-like cells
PBMCs	peripheral blood mononuclear cells
RXR	retinoid X receptor
T2DM	type 2 diabetes mellitus
TCR	T cell receptor
TNF-α	tumor necrosis factor alfa
TRAb	thyroid stimulating hormone receptor antibodies
VDR	vitamin D receptor
VDREs	vitamin D response elements
vNKT	variant Natural Killer T cells
